# Choroidal Detachment Secondary to Scleral Wound Leak After Ozurdex® Implantation in an Eye With Previous Vitrectomy: A Case Report

**DOI:** 10.7759/cureus.98542

**Published:** 2025-12-05

**Authors:** Sajad Hussain, Khawaja R Aziz, Ahmed Shehata

**Affiliations:** 1 Ophthalmology, Mid Yorkshire Trust, Wakefield, GBR; 2 Ophthalmology, Calderdale and Huddersfield NHS Trust, Huddersfield, GBR; 3 Ophthalmology, Calderdale Hospital, Huddersfield, GBR

**Keywords:** general internal medicine, medical retina and glaucoma, ophthalmology, ozurdex implant, rupture risk

## Abstract

Retinal vein occlusion (RVO) is a major cause of visual morbidity, and macular oedema secondary to RVO is increasingly managed with repeated dexamethasone intravitreal implant (Ozurdex^®^) therapy, particularly in eyes with suboptimal response to anti-vascular endothelial growth factor (VEGF) agents. While its efficacy is well established, the long-term cumulative risk to scleral integrity is less well characterised, especially in post-vitrectomy eyes. We report a case of an 86-year-old woman with a history of pars plana vitrectomy and recurrent macular oedema from branch RVO, treated over several years with repeated Ozurdex implants, who developed acute hypotony and choroidal detachment due to a scleral wound leak shortly after injection. Five days after implantation, the patient developed sudden vision loss and ocular pain with an intraocular pressure of 3 mmHg. Examination showed a positive Seidel test at the supertemporal entry site, and ultrasound revealed a non-kissing serous choroidal detachment. Urgent transconjunctival scleral suturing sealed the leak, resulting in rapid resolution of hypotony and anatomical recovery. Visual acuity improved from hand movements to 6/18 within one week, with subsequent stabilisation of macular oedema. A later steroid-related intraocular pressure rise was managed medically. This case emphasises how repeated injections into the same quadrant, combined with prior vitrectomy and age-related scleral changes, may predispose to focal scleral weakening, impaired wound closure, and hypotony. Early detection through Seidel testing and prompt surgical repair are crucial to preventing sight-threatening complications. Preventive measures, particularly injection-site rotation, careful post-injection wound assessment, and lower thresholds for suturing in high-risk eyes, are vital in long-term Ozurdex management.

## Introduction

Retinal vein occlusion (RVO) is a leading cause of visual impairment, with a high prevalence across Europe and a significant impact on long-term ocular health [1]. Macular oedema remains the main cause of vision loss in these patients, and intravitreal corticosteroid therapy is essential when anti-vascular endothelial growth factor (VEGF) response is inadequate, or the disease recurs [1]. 

The dexamethasone intravitreal implant (Ozurdex^®^) offers sustained anti-inflammatory effects via a biodegradable delivery system, and the landmark GENEVA trials confirmed its effectiveness in reducing oedema and improving visual outcomes in both branch and central RVO [2,3]. As many patients experience chronic or recurrent oedema, repeated Ozurdex implantation has become routine, supported by broader evidence of its success in other retinal inflammatory conditions [4-6].

However, the shift towards long-term, repeated steroid implant therapy has increased attention on the cumulative ocular risks. While ocular hypertension remains the most frequently discussed adverse event [7], emerging real-world studies suggest that structural complications, particularly those affecting scleral integrity and the development of hypotony, warrant closer scrutiny [7-9]. Previous research has shown that repeated transscleral interventions can alter scleral permeability and biomechanical strength [10,11], raising the possibility that repeated implantation could predispose certain eyes, especially those that have undergone vitrectomy or other surgical procedures, to wound leaks, choroidal detachment, or hypotony. Although hypotony after intravitreal injections is considered rare, its mechanisms, including reduced scleral rigidity, impaired wound healing, or inflammatory responses, are well understood and may be particularly relevant in cases involving repeated steroid implants [12].

Given the increasing reliance on repeated Ozurdex therapy for RVO management, only a small number of studies characterise the incidence, underlying mechanisms, and risk factors for hypotony in this setting. This case considers a patient with a vitrectomy on long-term Ozurdex who developed a scleral wound leak with hypotony and choroidal detachment.

## Case presentation

Ocular history

In the right eye (RE), her history includes meibomian gland dysfunction, blepharitis, dry eye, medial ectropion, and posterior capsule opacification, which was treated with a YAG capsulotomy.

The left eye (LE) has a more complex history. An epiretinal membrane (ERM) was diagnosed in 2015, followed by phacoemulsification with intraocular lens implantation, pars plana vitrectomy, ERM/ILM peel, and internal air tamponade in October 2015. Ectropion repair was subsequently performed in 2016 using a lateral tarsal strip, medial spindle, and punctoplasty. Since 2019, she has had a branched retinal vein occlusion (BRVO) with macular oedema, with a recurrence documented in 2023.

Prior and ongoing retinal therapy

Over the past five years, the LE has been treated with multiple Ozurdex implants after initial anti-VEGF therapy, including Eylea and previously ranibizumab. The most recent Ozurdex injection (22G) occurred before the adverse event on November 7, 2024. This was done at the same site previously used, the supratemporal. The only concurrent ophthalmic medications were topical lubricants (carmellose 0.5%, used four to six times daily in both eyes).

Status immediately before the event

At an RVO clinic visit in early March 2025, visual acuity in the LE was measured at 6/12, and OCT imaging demonstrated increased intraretinal fluid, exudates, and a persistent ERM. Additional OCT imaging continued to show worsening intraretinal fluid and exudates. Fluorescein angiography performed on March 20, 2025, revealed delayed filling, blood masking, microaneurysms, and central and peripheral capillary non-perfusion in the LE, consistent with deterioration of central macular oedema despite prior Ozurdex treatment.

Presentation and examination (March 25, 2025)

**Figure 1:**
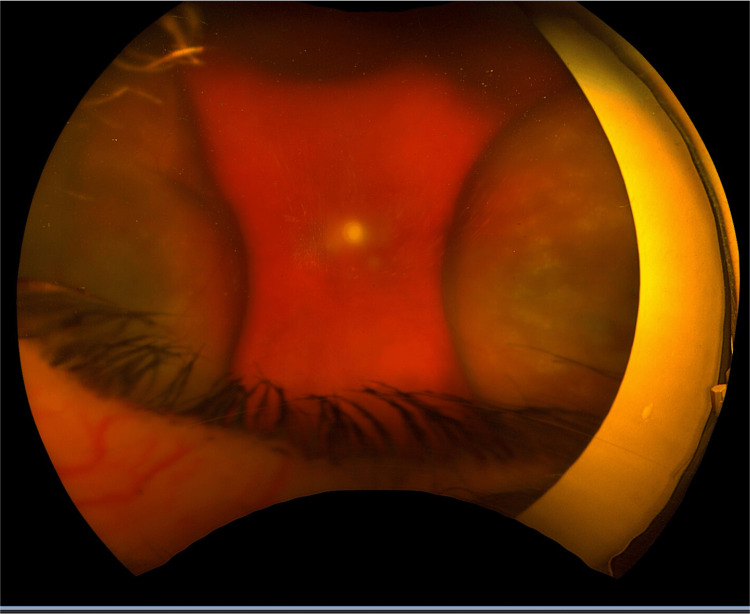
Fundus image of choroidal detachment

**Figure 2:**
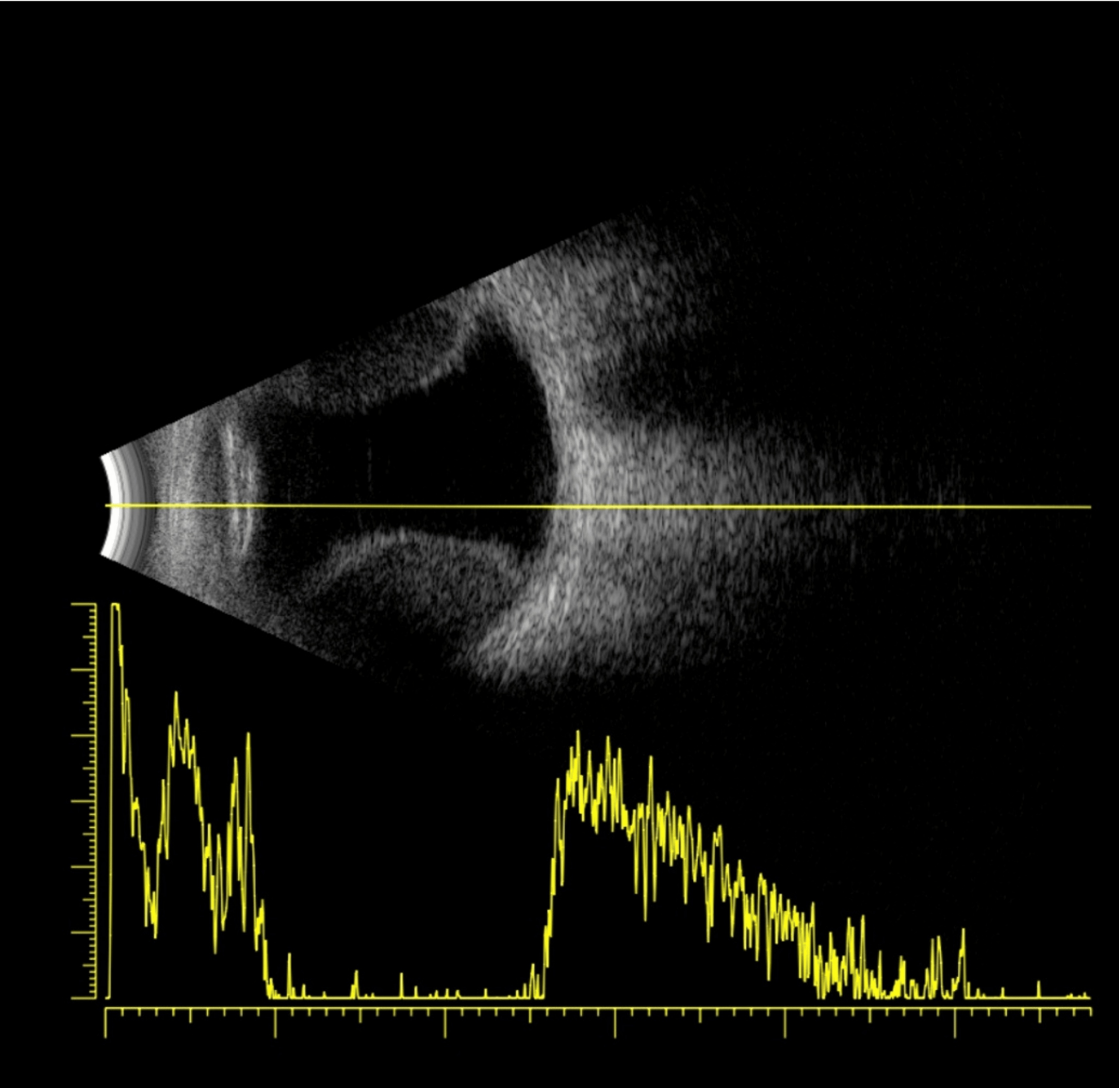
Ultrasound image showing choroidal detachment

On March 25, 2025, five days after an Ozurdex injection, the patient presented emergently with sudden vision loss in the LE, reduced to hand movements, accompanied by ocular pain and hypotony with an intraocular pressure of 3 mmHg. B-scan ultrasound showed a non-kissing choroidal detachment without evidence of retinal detachment. Slit-lamp examination revealed 2+ anterior chamber cells and a positive Seidel test at a superotemporal scleral entry site, indicating an active wound leak (Figures 1, 2).

Differential diagnosis

The differential diagnosis included post-injection endophthalmitis, which was ruled out due to rapid improvement, resolution of inflammation, and cessation of leakage after repair. Retinal detachment was excluded based on B-scan findings. Suprachoroidal haemorrhage was considered, but ultrasound favoured serous choroidal detachment. Medication-related hypotony was unlikely, as the patient was not on any hypotensive drops. The clinical picture most strongly supported a scleral wound leak with associated hypotony.

Management

Immediate surgical repair was undertaken under local anaesthesia. Two 8-0 Vicryl transconjunctival sutures were placed at the leaking superotemporal scleral site, after which the Seidel test became negative. A bandage contact lens was applied, and Maxitrol drops were initiated.

Post-operative course and follow-up

By March 28, 2025, visual acuity in the LE improved to 6/48, with a deep, quiet anterior chamber and resolving choroidal detachment. A mild residual leak persisted, and the bandage contact lens was replaced. At the vitreoretinal clinic on April 3, 2025, visual acuity improved to approximately 6/18 with pinhole correction, intraocular pressure rose to 12 mmHg, and the retina remained flat with the Ozurdex implant visible. At an RVO clinic review on May 8, 2025, visual acuity was 6/18 in the RE and 6/12 in the LE. Intraocular pressures measured 20 mmHg (RE) and 27 mmHg (LE). OCT showed improvement in macular oedema, and timolol 0.25% twice daily was started for the LE. The team planned to avoid the superotemporal quadrant for future interventions.

Outcome and follow-up

The patient demonstrated both anatomical and functional recovery over the following weeks. Visual acuity improved from hand movements to approximately 6/18, with complete resolution of the choroidal detachment and restoration of intraocular pressure to physiological levels. At six weeks, OCT confirmed improvement of macular oedema. A steroid-related rise in intraocular pressure was effectively controlled with topical timolol. The patient was counselled regarding injection-site rotation and the importance of promptly reporting post-injection pain or sudden vision changes.

## Discussion

Mechanism

This case reflects the interplay of focal scleral weakening and altered ocular biomechanics after vitrectomy. Scleral wound leakage following intravitreal injection represents a multifactorial process involving localised tissue stress, biomechanical fatigue, and compromised healing capacity. Repeated intravitreal injections into a single quadrant may induce localised microstructural disruption of collagen, reduced tensile strength, and localised thinning [12]. This can cause collagen remodelling and microstructural fatigue in scleral tissue, creating a “weak point” that is predisposed to wound dehiscence. In eyes with vitrectomy, the absence of vitreous body support alters intraocular fluid dynamics and scleral compliance, predisposing to inadequate self-sealing after intravitreal injection [13]. 

Vitrectomy itself can contribute to scleral weakening through iatrogenic incisions and postoperative remodelling [11]. When combined with chronic steroid exposure, these factors may lead to local modulation of extracellular matrix turnover, delayed fibroblast proliferation, and decreased scleral rigidity [11]. This constellation explains why our patient, elderly and vitrectomised, developed a focal wound leak shortly after repeat Ozurdex^®^ administration [11].

Hypotony after intravitreal injection most commonly arises from wound leakage, ciliary body shutdown, or choroidal effusion. The loss of vitreous tamponade can magnify the effect of even minor scleral defects, allowing egress of fluid, hypotony, and transudation across the choriocapillaris into the suprachoroidal space, culminating in serous choroidal detachment. The positive Seidel test and rapid resolution following suture closure confirm a wound-leak mechanism [13]. The subsequent serous choroidal detachment results from transudation of suprachoroidal fluid due to pressure imbalance, as seen in similar hypotony states [13]. 

Large-scale clinical trials such as the GENEVA trial by Haller et al. [3] and the HURON trial by Lowder et al. [4] demonstrated the excellent safety of Ozurdex^®^ in RVO and uveitis, respectively. They reported no incidence of hypotony. However, their strict inclusion criteria excluded eyes that had undergone previous vitrectomy, eyes with recent intraocular surgery, and eyes with thin sclera, thereby minimising exposure to wound-related complications. Consequently, clinical trials by Haller et al. [3] and the HURON trial by Lowder et al. [4] didn't observe any postoperative hypotony or choroidal detachments.

Subsequent real-world observational series report occasional wound complications in eyes receiving repeated dexamethasone implants, particularly in cases with previous vitrectomies performed or those with scleral thinning [5,6]. Small-sample studies done by Maggio et al. [8] and Pommier et al. [9] suggest that wound leakage is under-recognised because subtle hypotony may go undetected if not actively tested by Seidel or tonometry in the early post-injection phase. Studies of Ozurdex in eyes with prior vitrectomy typically report favourable outcomes without prominent wound-leak signals, but cohorts often involve single or few injections; Adán et al. [6] reported that wound leaks occur as a late sign, generally after multiple dexamethasone implants in eyes with previous vitrectomy, which aligns with our patient’s course. 

Practical implications

Rotation of injection sites and quadrant planning should be employed to prevent repeated scleral entry at the same clock hour, alternating between superior-temporal and inferotemporal sites or across meridians, especially in patients with thin sclera, older age, or post-vitrectomy status. After Ozurdex delivery, rigorous aseptic technique must be coupled with direct wound inspection to identify any shelled or gaping sclerotomy; if uncertainty remains, a temporary pressure patch, bandage contact lens, or immediate suture application may be appropriate. Clinicians should remain highly vigilant for hypotony, ensuring patients are informed to report pain or visual blurring within one to seven days post-injection, as a simple Seidel test can swiftly confirm leakage. When necessary, early surgical closure should be performed because prompt repair reverses hypotony and prevents complications such as choroidal apposition, maculopathy, or hypotony-related optic neuropathy. Post-repair follow-up should include monitoring for biphasic intraocular pressure behaviour, with an initial return to normal levels followed by a possible steroid-induced rise, requiring timely intraocular pressure checks and topical therapy as needed.

Limitations

This report describes a single-patient observation. Scleral microanatomy was not measured using imaging techniques such as anterior-segment OCT or ultrasound. Nevertheless, the consistent temporal sequence, objective Seidel positivity, and total resolution after repair offer strong mechanistic evidence. 

Patient consent

Verbal and written informed consent for publication of clinical details and images was obtained from the patient.

## Conclusions

This case demonstrates that scleral wound leakage with secondary hypotony and choroidal detachment can occur in eyes with previous vitrectomy, following repeated dexamethasone implant injections. The pathogenesis involves cumulative scleral stress, absence of vitreous tamponade, and age-related collagen weakening. Early diagnosis via Seidel testing and prompt surgical closure restores intraocular pressure, prevents choroidal apposition, and averts permanent visual damage. Future preventive strategies should include rotating injection quadrants, careful wound inspection, and lower thresholds for suture placement in high-risk patients. Ultimately, this case highlights the importance of individualised injection planning and vigilance in long-term Ozurdex^®^ therapy, especially in the elderly or patients with previous vitrectomy.
